# Let-7 suppresses liver fibrosis by inhibiting hepatocyte apoptosis and TGF-β production

**DOI:** 10.1016/j.molmet.2023.101828

**Published:** 2023-10-28

**Authors:** Jiahui Song, Haining Lv, Beibei Liu, Mingjun Hao, Hugh S. Taylor, Xuchen Zhang, Da Li, Yingqun Huang

**Affiliations:** 1 Center of Reproductive Medicine, National Health Commission Key Laboratory of Advanced Reproductive Medicine and Fertility, Shengjing Hospital of China Medical University, Shenyang 110004, China; 2 Department of Obstetrics, Gynecology and Reproductive Sciences, Yale University School of Medicine, New Haven, CT 06520, USA; 3 Department of Obstetrics and Gynecology and Center for Reproductive Medicine, Affiliated Drum Tower Hospital, Medical School of Nanjing University, Nanjing 210008, China; 4 Department of Pathology, Yale University School of Medicine, New Haven, CT 06510, USA; 5 Yale Center for Molecular and Systems Metabolism, Yale University School of Medicine, New Haven, CT 06520, USA

**Keywords:** Fibrosis, Let-7, Apoptosis, Gene therapy, Liver, AAV, miRNA

## Abstract

**Objective:**

FAS-mediated apoptosis of hepatocytes and aberrant TGF-β signaling are major drivers of liver fibrosis. Decreased miRNA let-7 expression in the livers of patients and animals with fibrosis suggests a mechanistic link of let-7 to hepatic fibrogenesis.

**Methods:**

Using transient transfection we tested the effects of let-7 overexpression and TET3 siRNA knockdown on FAS and TGF-β1 expression and FAS-mediated apoptosis in human and mouse primary hepatocytes. We assessed the therapeutic activity of let-7 miRNA delivered via adeno-associated viral vectors in mouse models of carbon tetrachloride (CCl_4_)-induced and bile duct ligation (BDL)-induced liver fibrosis.

**Results:**

Let-7 decreased TGF-β1 production from hepatocytes through a negative feedback loop involving TET3. On the other hand, let-7 post-transcriptionally inhibits FAS expression, thereby suppressing hepatocyte apoptosis. Hepatic-specific delivery of let-7 miRNA mitigated liver fibrosis in both CCl_4_ and BDL mouse models.

**Conclusions:**

Let-7 is a crucial node in the signaling networks that govern liver fibrosis progression. Let-7 and/or its derivatives may be used as therapeutic agents for liver fibrosis.

## Highlights

• Let-7 performs a dual role in repression of hepatic fibrosis.
• Let-7 inhibits TGF-β production through a let-7/TET3 negative feedback mechanism.
• Let-7 suppresses FAS-mediated apoptosis of hepatocytes.
• Liver-specific delivery of let-7 ameliorates fibrosis in two murine models with different etiologies.

## 1 Introduction

Hepatic fibrosis is the formation of fibrous scars that result from chronic hepatocellular damage caused by, for example, alcohol abuse, hepatitis B/C (HBV/HCV) infection, biliary obstruction, or nonalcoholic steatohepatitis (NASH). Liver fibrosis predisposes patients to cirrhosis, liver failure and hepatocellular carcinoma (HCC), and is a major cause of morbidity and mortality worldwide [1]. Studies of patients and rodent models of liver fibrosis have identified common key molecular mechanisms leading to fibrosis. These include hepatic oxidative stress, FAS-mediated apoptosis of hepatocytes, activation of hepatic stellate cells (HSCs), release of TGF-β by hepatocytes and HSCs, and excessive production of extracellular matrix (ECM) from HSCs [[1], [2], [3], [4], [5]]. Currently, no effective therapies are available for liver fibrosis. An improved understanding of cellular and molecular pathways promoting fibrosis is of paramount importance for the development of effective treatments for the disease.

The TET family of dioxygenases (TET1, TET2 and TET3) oxidize 5-methylcytosines (5 mC) to 5-hydroxymethylcytosines (5hmC) and its derivatives to mediate DNA demethylation [[6], [7], [8]]. We have previously shown that TGF-β upregulates TET3 expression and TET3 in turn epigenetically stimulates TGF-β expression and that this TET3/TGF-β positive feedback regulation occurs both in hepatocytes and HSCs [9]. Importantly, hepatic delivery of siRNAs specifically targeting TET3 using adeno-associated viruses serotype 8 (AAV8) ameliorated fibrosis in a mouse model of carbon tetrachloride (CCl_4_)-induced liver fibrosis, demonstrating the TET3/TGF-β positive feedback loop as a crucial mechanism of liver fibrosis [9].

The human and mouse *TET3*/*Tet3* mRNAs contain multiple binding sites for miRNA let-7 and are targets of let-7-mediated inhibition at the posttranscriptional level [[10], [11], [12]]. We have reported aberrantly increased expressions of TET3 in hepatocytes of obesity, diabetes and liver fibrosis and that hepatic oxidative stress downregulates let-7 leading to de-repression of TET3 [11]. Hepatic oxidative stress occurs in obesity, diabetes and liver fibrosis [4,9,[11], [12], [13], [14], [15]]. Curiously, an inverse relationship between circulating let-7 levels and the severity of hepatic fibrosis in patients has been documented [16,17]. Likewise, levels of let-7 were found to significantly decrease in liver tissues and blood samples from patients with hepatic fibrosis as well as from mice with CCl_4_-induced liver fibrosis or diet-induced NASH [18,19]. However, whether decreased hepatic let-7 expression plays a causal role in fibrosis and if so, what could be the underly mechanism, have not been determined.

In the current study, we use two murine models of liver fibrosis induced by CCl_4_ and bile duct ligation (BDL), respectively, to demonstrate that liver-specific delivery of let-7 using AAV8 ameliorates fibrosis. Mechanistically, we show that let-7 in hepatocytes performs a dual role in repression of fibrogenesis: by inhibiting TGF-β production through a novel let-7/TET3 negative feedback regulation and by suppressing FAS-mediated apoptosis of hepatocytes.

## 2 Materials and methods

### 2.1 Mouse

All animal work was approved by the Animal Ethics Committee of Shengjing Hospital of China Medical University in accordance with the guidelines of the Experimental Animal Regulation by the National Science and Technology Commission, China. Male C57BL/6J mice were purchased from Beijing Vital River Laboratory Animal Technology and housed at 22°C–24 °C with a 12 h light/12 h dark cycle with regular chow (Harlan Teklad no. 2018, 18 % calories from fat) and water provided ad libitum. Before experiments, mice were allowed to acclimate for at least 7 days in the animal facility.

### 2.2 In vivo virus administration

The AAV8-let-7a (GFPmmu-let-7a-5p AAV miRNA virus serotype 8, Amm1000108) and AAV8-vec (empty vector control virus serotype 8, Am00100) were purchased from Applied Biological Materials Inc. (also refer to Table S2 in the Supplementary Information). Mice were tail vein injected with AAV8-let-7a or AAV8-vec once a week at week 3, 4 and 5 (Figure 2A) or week 2, 3 and 4 (Figure 3A). All viruses were injected at 2 × 10^10^ gc/mouse in 150 μl of DPBS (calcium/magnesium-free, Gibco, 14190144). The tail was cleansed with 70 % ethanol and the injection was made in the lateral vein, using 30-gauge needles.

**Figure 1:**
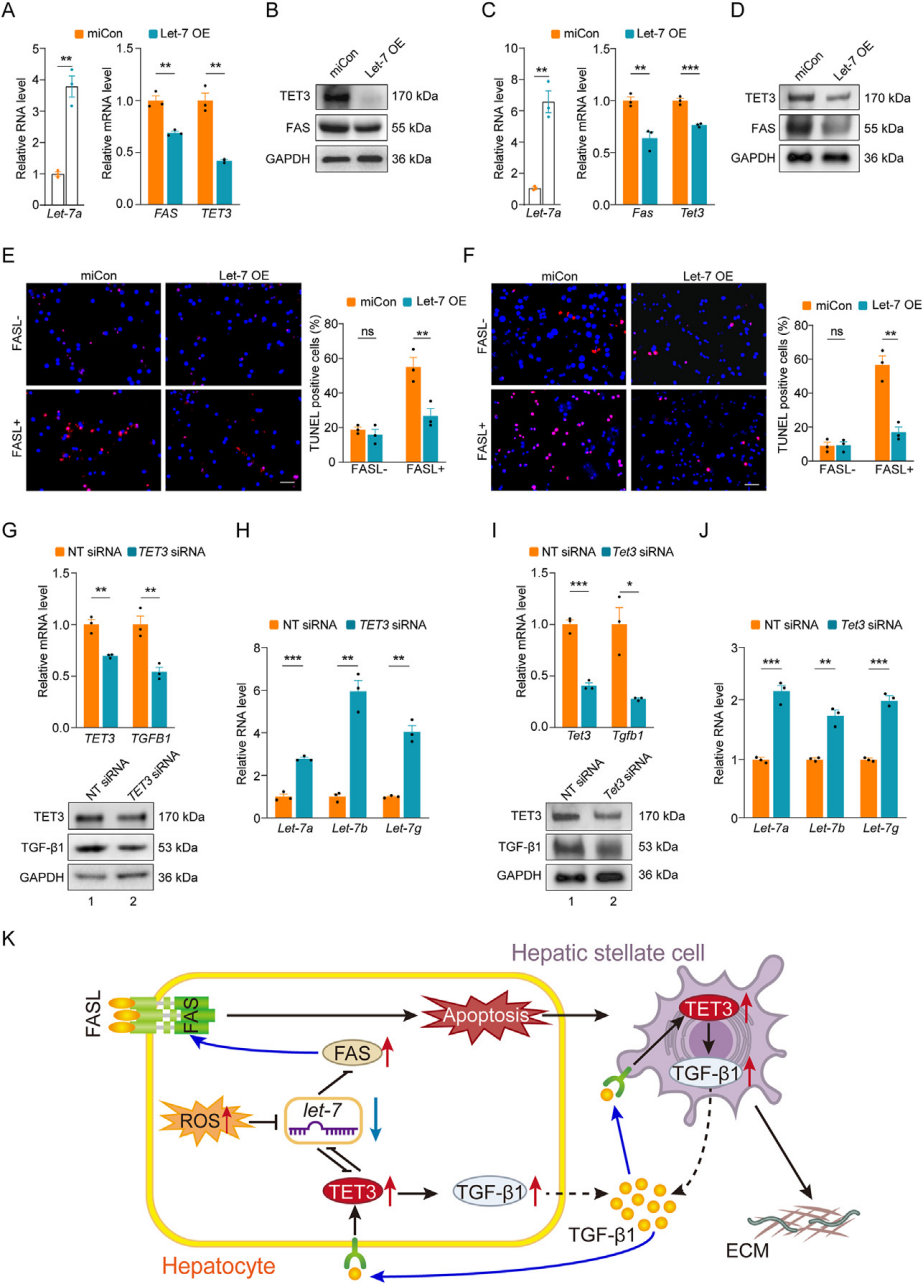
**Let-7-mediated regulation of FAS and apoptosis in primary hepatocytes from obese humans and mice. A**) Left panel, qPCR of *let-7a* from human hepatocytes transfected with non-targeting control miRNA (miCon) or *let-7a* for 24 h *n* = 3 per group in technical replicates. Right panel, qPCR of *TET3* and *FAS* mRNAs from human hepatocytes transfected with miCon or *let-7a* for 24 h *n* = 3 per group in technical replicates. **B**) Representative immunoblots for TET3 and FAS from human hepatocytes treated as in **A**. Proteins were isolated at 48 h post-transfection. GAPDH was used as a loading control. Protein sizes in kDa are marked on the right. **C**) Left panel, qPCR of *let-7a* from mouse hepatocytes transfected with miCon or *let-7a* for 24 h *n* = 3 per group in technical replicates. Right panel, qPCR of *Tet3* and *Fas* mRNAs from mouse hepatocytes transfected with miCon or *let-7a* for 24 h *n* = 3 per group in technical replicates. **D**) Representative immunoblots for TET3 and FAS from mouse hepatocytes treated as in **C**. Proteins were isolated at 48 h post-transfection. **E**) Human hepatocytes were treated as in **A**. TUNEL assays were performed at the 48 h time point. Representative photomicrographs and corresponding statistical analysis of TUNEL^+^ (red) cells showing a significant decrease in FASL-dependent apoptotic death in let-7a-transfected as compared to miCon-transfected cells. FASL-, no FASL added; FASL+, FASL added. *n* = 3 randomly selected areas per group. **F**) Mouse hepatocytes were treated as in **C**. TUNEL assays were performed at 48 h time point. Representative photomicrographs and corresponding statistical analysis of TUNEL^+^ (red) cells showing a significant decrease in FASL-dependent apoptotic death in let-7a-transfected vs. miCon-transfected cells. *n* = 3 randomly selected areas per group. **G**) Top panel: qPCR of *TET3* and *TGFB1* mRNAs from human hepatocytes transfected with non-targeting control siRNA (NT siRNA) or siRNA specifically targeting human *TET3* (*TET3* siRNA) for 24 h *n* = 3 per group in technical replicates. Bottom panel: representative immunoblots for TET3 and TGF-β1 from human hepatocytes. Proteins were isolated at 48 h post-transfection. **H**) qPCR of let-7a, let-7b and let-7g from human hepatocytes treated as in **G**. *n* = 3 per group in technical replicates. **I**) Top panel: qPCR of *Tet3* and *Tgfb1* mRNAs from mouse hepatocytes transfected with NT siRNA or siRNA specifically targeting mouse *Tet3* (*Tet3* siRNA) for 24 h *n* = 3 per group in technical replicates. Bottom panel: representative immunoblots for TET3 and TGF-β1 from mouse hepatocytes. Proteins were isolated at 48 h post-transfection. **J**) qPCR of let-7a, let-7b and let-7g from mouse hepatocytes treated as in **I**. *n* = 3 per group in technical replicates. Error bars are mean with SEM of technical replicates. ∗*P* < 0.05, ∗∗*P* < 0.01, and ∗∗∗*P* < 0.001, by 2-tailed Student's *t* test. All data are representative of at least two independent transfection experiments. Scale bar: 50 μm. **K**) A let-7-mediated mechanism of fibrogenesis. Oxidative stress-induced downregulation of let-7 expression in hepatocytes leads to de-repression of TET3 and FAS. Signals emanated from apoptotic hepatocytes activate HSCs which in turn enhance the production of TGF-β1 and ECM. On the other hand, TGF-β1 released from hepatocytes and HSCs as a result of increased TET3 expression acts as both an autocrine and paracrine factor to stimulate TET3 expression, forming a positive feedback loop of TET3 and TGF-β1. Finally, the negative feedback regulation between TET3 and let-7 in hepatocytes exacerbates the vicious cycle of hepatocyte apoptosis, HSC activation, TGF-β1 release and ECM production.

**Figure 2:**
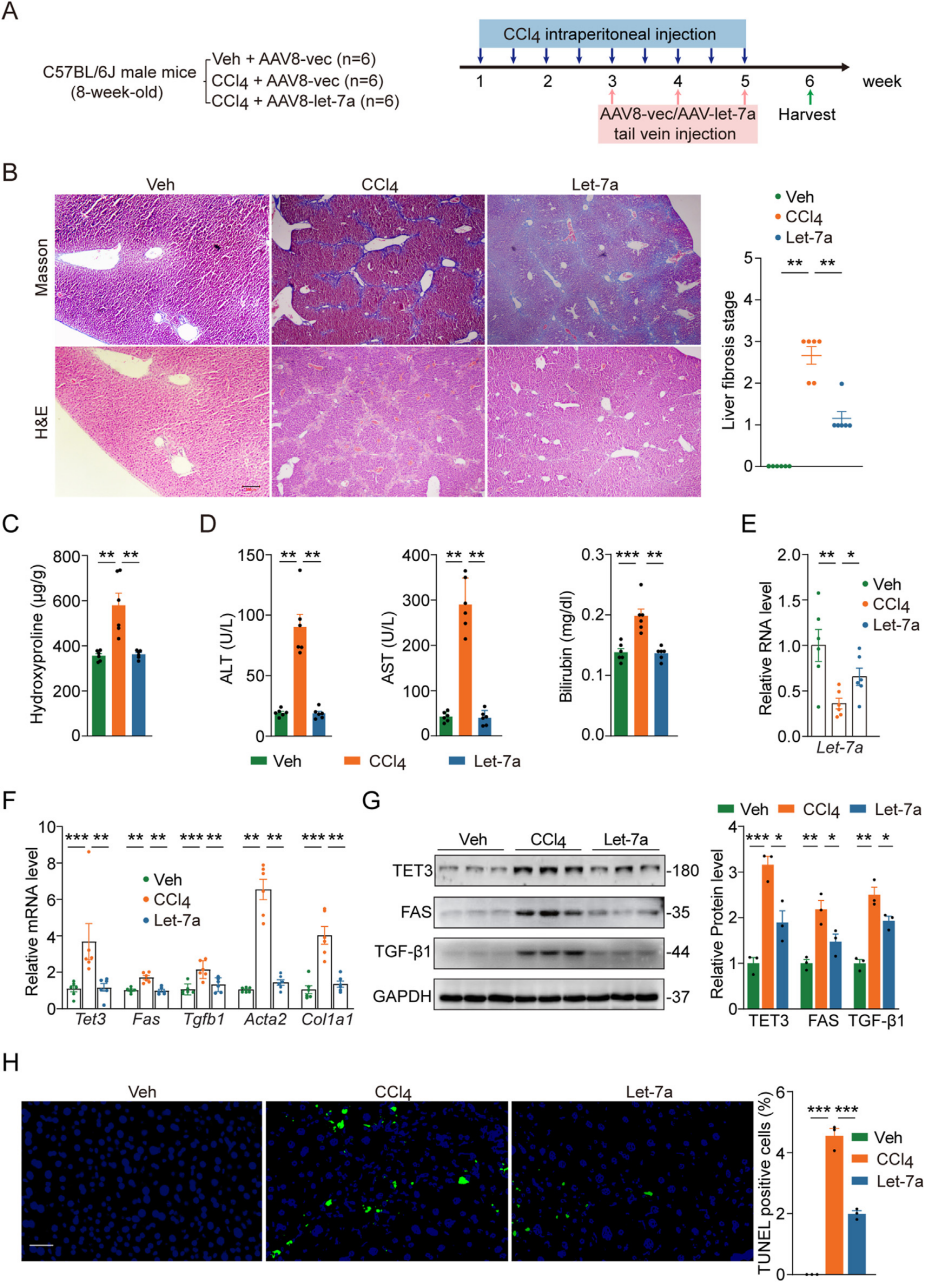
**Effects of let-7 on CCl**_**4**_**model. A**) Schematic of experimental design. **B**) Representative images of Masson's trichrome and H&E staining on liver sections from mice subjected to the indicated treatments, with quantification of fibrosis stage shown on the right. Scale bar: 200 μm. **C**) Hydroxyproline contents from liver tissues isolated from mice treated as in **A**. **D**) Plasma ALT, AST and bilirubin from mice treated as in **A**. **E**) qPCR of Let-7a from liver tissues isolated from mice treated as in **A**. **F**) qPCR of indicated genes from liver tissues isolated from mice treated as in **A**. **G**) Representative immunoblots for TET3, FAS and TGF-β1 of liver tissues from mice treated as in **A**, with quantification shown on the right. **H**) TUNEL assays on liver sections from mice treated as in **A**. Representative photomicrographs and corresponding statistical analysis of TUNEL^+^ (green) cells are presented. *n* = 3 randomly selected areas per group. Scale bar: 40 μm. In **B**–**F**, each data point represents an individual mouse, with 6 mice in each group. In **G**, each data point represents an individual mouse, with 3 mice in each group. Error bars are mean with SEM. ∗*P* < 0.05, ∗∗*P* < 0.01 and ∗∗∗*P* < 0.001, by one-way ANOVA with Tukey post-test.

**Figure 3:**
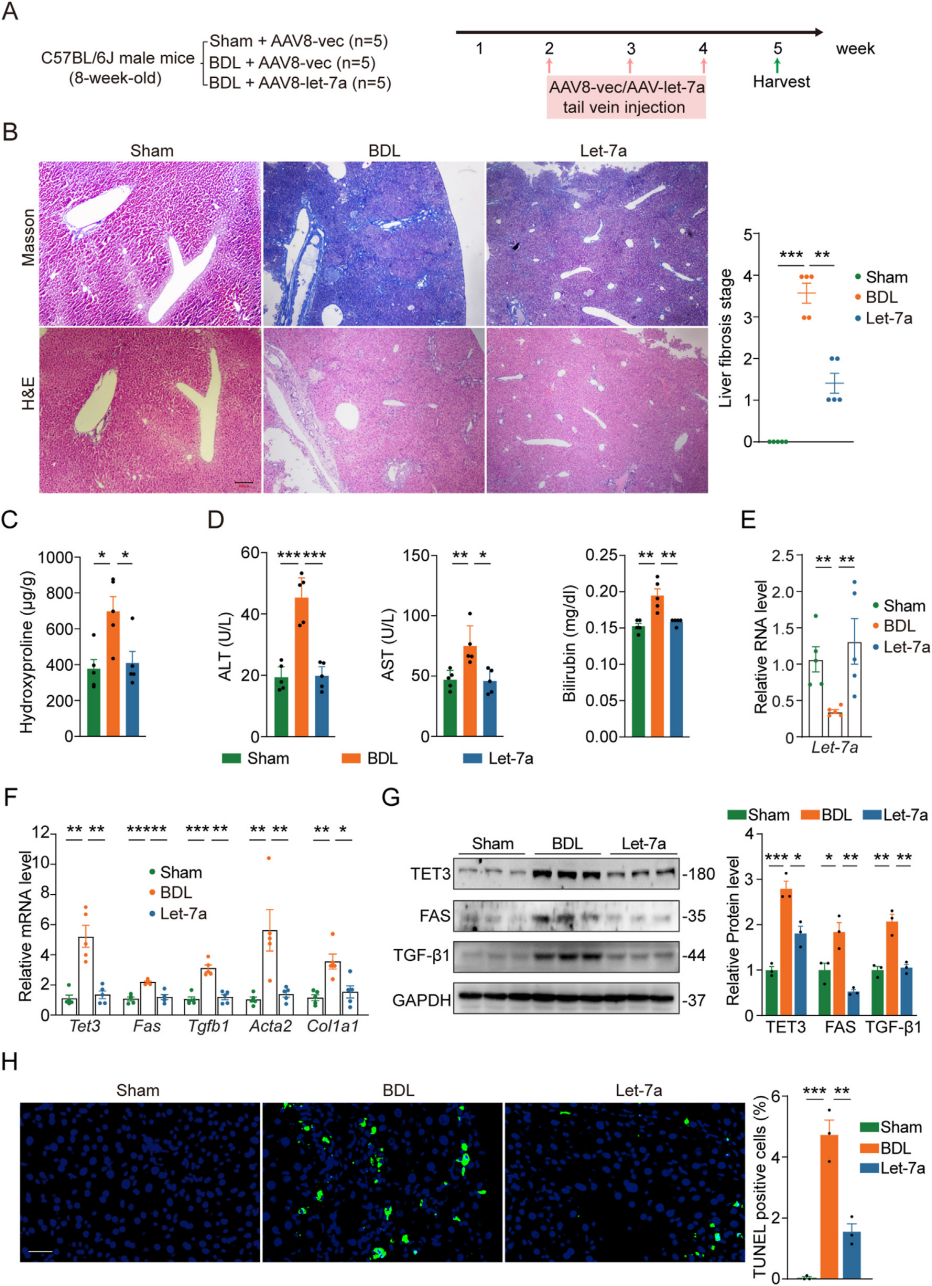
**Effects let-7 on BDL model. A**) Schematic of experimental design. **B**) Representative Masson's trichrome and H&E staining on liver sections from mice subjected to the indicated treatments, with quantification of fibrosis stage shown on the right. Scale bar: 200 μm. **C**) Hydroxyproline contents from liver tissues isolated from mice treated as in **A**. **D**) Plasma ALT, AST and bilirubin from mice treated as in **A**. **E**) qPCR of Let-7a from liver tissues isolated from mice treated as in **A**. **F**) qPCR of indicated genes from liver tissues isolated from mice treated as in **A**. **G**) Representative immunoblots for TET3, FAS and TGF-β1 of liver tissues from mice treated as in **A**, with quantification shown on the right. **H**) TUNEL assays on liver sections from mice treated as in **A**. Representative photomicrographs and corresponding statistical analysis of TUNEL^+^ (green) cells are presented. *n* = 3 randomly selected areas per group. Scale bar: 40 μm. In **B**–**F**, each data point represents an individual mouse, with 5 mice in each group. In **G**, each data point represents an individual mouse, with 3 mice in each group. Error bars are mean with SEM. ∗*P* < 0.05, ∗∗*P* < 0.01 and ∗∗∗*P* < 0.001, by one-way ANOVA with Tukey post-test.

### 2.3 Mouse liver fibrosis induction and treatment

For CCl_4_ model, mice were randomly divided into three groups: control (Veh + AAV8-vec), model (CCl_4_ + AAV8-vec), and treatment (CCl_4_ + AAV8-let-7a). To induce fibrosis, male mice at 8 weeks of age were intraperitoneally injected with mineral oil (Sigma, M5310) or 10 % CCl_4_ (Sigma, 319961, dissolved in mineral oil) at 0.6 ml/kg of body weight twice a week from week 1 through week 5. Tail vein injections of AAV8-let-7a or AAV8-vec viruses were performed weekly on week 3, 4, and 5. On week 6, blood and livers were collected for histological, blood chemistry, and molecular readouts of fibrosis. For BDL model, mice were randomly divided into three groups: group 1 (Sham + AAV8-vec), group 2 (BDL + AAV8-vec), and group 3 (BDL + AAV8-let-7a). Surgeries were performed on week 1 to induce obstructive cholestatic liver injury or sham as previously described [20]. Intraperitoneal injections of AAV8-vec or AAV8-let-7a viruses were performed on week 2, 3, and 4. Blood and liver tissues were harvested on week 5.

### 2.4 Histopathology examination

Mouse liver samples were collected and fixed at 4 °C with 4 % of phosphate buffered paraformaldehyde for 24 h. Fixed tissue specimens were embedded in paraffin and cut into 4 μm-thick tissue sections for histopathological analysis. Tissue sections were stained with Hematoxylin-Eosin or Masson Trichrome according to standard instructions. The stained slides were examined using a light microscope by two experienced hepatologists who were blinded to the study protocol. The stage of liver fibrosis was assessed based on the following criteria: 0, no fibrosis; 1, fibrosis present with collagen fibers extending from the portal triad/central vein to peripheral regions; 2, mild fibrosis with the formation of fibrous septae but with intact architecture of the liver lobules; 3, moderate fibrosis with fibrous septum accompanied by intralobular structural disorders but without cirrhosis; and 4, definite cirrhosis.

### 2.5 Blood chemistry

Blood samples were collected in EDTA tubes (Microtainer with K_2_EDTA, BD, 365974) by cardiac puncture of terminally anesthetized animals. The tubes were centrifuged at 2,000×*g* at 4 °C for 20 min, and supernatant plasma was collected and stored at −80 °C until use. Kits used to measure alanine transaminase (EALT-100) and aspartate transaminase (EASTR-100) were purchased from Bioassay Systems. The bilirubin assay kit (MAK126) and Hydroxyproline Assay Kit (MAK008-1 KT) were purchased from Sigma Aldrich.

### 2.6 Primary hepatocytes and transfection

Primary hepatocytes were seeded in 12- or 24-well plates (Corning BioCoat Cellware, Collagen Type I, 62405-607). Mouse primary hepatocytes were prepared as previously described [12] and maintained in a complete culture medium (Williams’ Medium [GIBCO,12551, contains 11 mM glucose] supplemented with 5 % FBS, 10 mM HEPES buffer [GIBCO, 15630-080], 2 mM L-Glutamine [GIBCO, 25030-081], 1 % SPA [GIBCO, 15240-062], 4 mg/L insulin [GIBCO, 12585-014] and 1 μM dexamethasone [Sigma, D4902]). Cryoplateable primary human hepatocytes (HHA1000-IV210000097060718V1, lot# HH1137, 57 years old caucasian female, BMI 37; HHA1000-IV210000118111917V1, Lot# HH1161, 35 years old African American male, BMI 32) were purchased from Discovery Life Sciences. Cells were thawed using 37 °C Universal Cryopreservation Recovery Media (UCRM, 81015, Discovery Life Sciences) and seeded in Universal Primary Cell Plating Media (UPCM, 81016, Discovery Life Sciences).

### 2.7 Transfection of primary hepatocytes

For let-7a transfection experiments, cells were seeded in 24-well (or 12-well) plates at a density of 2 × 10^5^ (or 4 × 10^5^) cells/well for mouse primary hepatocytes and 3.5 × 10^5^ (or 7 × 10^5^) cells/well for human primary hepatocytes the day before transfection. To prepare let-7a transfection solution for each 24-well of cells, 3 pmol of miCon or let-7a were mixed with 15 μl of OPTI-MEM (Gibco，31985-070) by gentle pipetting. In parallel, 1 μl of Lipofectamine RNAiMAX (Invitrogen, 13778-150) was mixed with 15 μl of OPTI-MEM. Following 5 min of incubation at room temperature (RT), the two contents were mixed by gentle pipetting and the resulting 30 μl of transfection solution was added to one well of cells containing 1 ml culture media. Media were changed the next day, followed by analyses at the time points indicated in the figure legends. For siRNA transfection in a 24-well plate scale, 3 pmol NT siRNA (AM4636, Ambion), *Tet3* siRNA (mouse, 4390815/s101483, Ambion) or *TET3* siRNA (4392420/s47239, Ambion) was mixed with 15 μl of OPTI-MEM by gentle pipetting. In parallel, 1 μl of Lipofectamine RNAiMAX was mixed with 15 μl of OPTI-MEM by gentle pipetting. Following 5 min of incubation at RT, the resulting 30 μl of transfection solution was added to each well of cells containing 1 ml of culture media. Media were changed the next day, followed by analyses at the time points indicated in the figure legends.

### 2.8 RNA extraction and RT-qPCR assays

Total RNA was extracted from primary hepatocytes or liver tissue samples using PureLink RNA Mini Kit (Ambion, 12183018A). For mRNA PCR, 0.8 μg of total RNA was reverse transcribed to cDNA in a reaction volume of 20 μl using PrimeScript RT Reagent Kit (TAKARA, RR037A). Quantitative real-time PCR reactions were carried out using iQSYBRGreen (Bio-Rad) in a Bio-Rad iCycler. Gene expression levels were normalized against RPLP0. The specific PCR primers for mouse and human were summarized in Supplementary Table S1. For let-7 PCR, 0.8 μg of total RNA was reverse transcribed to cDNA in a reaction volume of 20 μl using miScript II RT kit (QIAGEN, 218161). Quantitative real-time PCR reactions were carried out using miScript SYBR Green PCR Kit (QIAGEN, 218073) in a Bio-Rad iCycler. Gene expression levels were normalized against RNU6-2-11. The specific PCR primers were summarized in Supplementary Table S1.

### 2.9 Western blot analysis

Primary hepatocytes were homogenized in situ using a pipette tip in 2x SDS-sample buffer with 10 % β-mercaptoethanol at RT in less than 5 s followed by heating at 100 °C for 5 min with occasional vortexing. Samples were loaded at 5–10 μl per well onto a 4–15 % gradient SDS gel (Bio-rad), followed by Western blot analysis. The antibodies used were anti-TET3 (for mouse TET3, dilution 1:1000, Active motif, 61395), anti-TET3 (for human TET3, dilution 1:1000, GeneTex, GTX121453), anti-FAS (dilution 1:1000; Proteintech, 60196-1-Ig), anti-TGF-β1 (dilution 1:400; Proteintech, 21898-1-AP), and HRP-conjugated anti-GAPDH (dilution 1:5000; Proteintech, HRP-60004). The secondary antibody was HRP-linked Anti-rabbit IgG (dilution 1:10,000; Rockland, 611-1322). Protein quantification was performed using ImageJ software.

### 2.10 TUNEL assay

Human recombinant fas ligand (FASL, Sigma Aldrich, S8689) were reconstituted with sterile water to a concentration of 0.1 mg/mL and further diluted with PBS containing 0.1 % bovine serum albumin. After transfection with miCon or let-7a for 32 h, cells were treated with 1 ng/mL FASL or the same volume of PBS containing 0.1 % bovine serum albumin in the presence of miCon or let-7a. After 16 h, TUNEL assays were performed using the *In Situ* Cell Death Detection Kit (Sigma Aldrich, 12156792910). Briefly, cells were fixed with 4 % paraformaldehyde in PBS for 1 h at RT and rinsed with PBS, followed by permeabilization with 0.1 % Triton X-100 in 0.1 % sodium citrate for 2 min on ice. TUNEL reaction mixture was added and incubation was carried out at 37 °C for 60 min. DAPI was added to counter-stain the cells for 1 min. The slides were coverslipped and scoped using a Keyence BZ-X700 fluorescence microscope. TUNEL staining of mouse liver FFPE slides was performed using One-step TUNEL Assay Kit (E-CK-A320 (Elabscience) to detect apoptotic cells according to the manufacturer's instructions. Images were captured using a Keyence BZ-X700 fluorescence microscope.

### 2.11 Statistical analysis

The number of independent experiments and the statistical analysis for each figure are indicated in the legends. All statistical analyses were performed using GraphPad Prism version 8 for Windows (GraphPad Software, La Jolla California USA, www.graphpad.com) and are presented as mean ± SEM. Comparisons between two groups were done by Two-tailed Student's *t* tests. Comparisons among three groups were done using one-way ANOVA with Tukey post-test. *p* < 0.05 was considered significant.

## 3 Results

### 3.1 Let-7 inhibits FAS expression in hepatocytes

FAS is a member of the TNF-death receptor family. Upon binding by FAS ligand (FASL) which is mainly expressed by T cells and natural killer cells, FAS is activated leading to apoptotic cell death. Abnormally increased expressions of FAS and FASL have been documented in inflammatory liver diseases including fibrosis [2]. In cancer and immune cells let-7 targets the 3’ untranslated regions (UTRs) of human and mouse *FAS*/*Fas* mRNAs thereby inhibiting their expression at the post-transcriptional level [[21], [22], [23]]. To test whether let-7 inhibits FAS expression in hepatocytes, we transfected a single let-7 isoform, let-7a, into primary hepatocytes isolated from obese humans and mice known to have decreased let-7 expression due to hepatic oxidative stress [11]. mRNA target recognition by miRNAs is mainly determined by “seed” sequences, i.e., nucleotides 2-7 in mature miRNAs. All mature let-7 family members share the same seed sequence [24]. When let-7a was overexpressed in obese human hepatocytes (Figure 1A, left panel), we observed decreased TET3 expression both at the mRNA (Figure 1A, right panel) and protein (Figure 1B, top blot) levels. The expression of FAS was also inhibited by let-7a overexpression (Figure 1A, right panel; Figure 1B, middle blot). Similar results were obtained in primary hepatocytes from obese mice (Figure 1C,D). Notably, the inhibition of TET3 by let-7 was much more pronounced in human hepatocytes than that in mouse hepatocytes (Figure 1B,D). We hypothesized that TET3 mRNA or TET3 protein in human hepatocytes is less stable than those in mouse hepatocytes. Collectively, we conclude that let-7 inhibits the expression of TET3 and FAS in hepatocytes.

### 3.2 Let-7 inhibits FAS-mediated apoptosis of hepatocytes

Hepatocytes are the major parenchymal cells of the liver. Signals elicited from apoptotic hepatocytes, including reactive oxygen species (ROS), hedgehog ligands and nucleotides, activate HSCs and drive inflammation and fibrogenesis [25]. As let-7 inhibits FAS expression (Figure 1A–D), we asked whether let-7 overexpression would suppress FAS-mediated cell death. Indeed, we observed a significant decrease in FAS-dependent apoptosis of human (Figure 1E) and mouse (Figure 1F) hepatocytes transfected with let-7a. Thus, let-7 suppresses apoptosis of hepatocytes at least in part by downregulating FAS expression.

### 3.3 Let-7 expression is negatively regulated by TET3

Given that let-7 inhibits TET3 expression (Figure 1A–D), we tested whether TET3 and let-7 might reciprocally regulate each other. Thus, we reduced TET3 expression using siRNAs in primary hepatocytes from obese humans and mice. We specifically tested three let-7 isoforms, let-7a, let-7b and let-7g, as these have been shown to decrease in liver tissues and blood samples from humans and mice with NASH/liver fibrosis [[16], [17], [18], [19]]. When TET3 was downregulated at the mRNA (Figure 1G, top panel) and protein (Figure 1G, bottom panel) levels in human hepatocytes, we observed increased expression of all three let-7 isoforms (Figure 1H). Similar results were obtained in mouse hepatocytes (Figure 1I,J). These results are consistent with inhibition of let-7 expression by TET3, though the underlying mechanism of this inhibition warrants future investigation. Based on these results, we suggest a negative feedback regulation between let-7 and TET3 in hepatocytes, a discovery not previously documented.

### 3.4 A proposed let-7-mediated mechanism

We have previously shown that TET3 stimulates TGF-β expression in HSCs as well as hepatocytes isolated from healthy humans and mice [9]. Mechanistically, TET3 binds to and induces demethylation of the *Tgfb1* promoter, upregulating transcription [9]. When TET3 was downregulated by siRNA in hepatocytes from obese humans, the expression of TGF-β1 was decreased both at the mRNA (Figure 1G, Top panel) and protein (Figure 1G, bottom panel) levels. Similar results were observed in hepatocytes from obese mice (Figire 1I,J). Thus, a positive regulation of TGF-β expression by TET3 also occurs in hepatocytes from obese humans and mice. We have previously documented that exposing mice to a high fat-diet (HFD) induces hepatic oxidative stress leading to decreased let-7 expression [11]. We have also reported that there exists a positive feedback regulation between TET3 and TGF-β both in hepatocytes and HSCs [9]. Thus, we propose a novel let-7-mediated mechanism in liver fibrogenesis illustrated in Figure 1K.

### 3.5 Let-7 attenuates CCl_4_-induced liver fibrosis

Let-7 expression is decreased in the livers of patients and rodents with liver fibrosis [18,19]. Given our newly discovered mechanism of let-7 (Figure 1K), we tested the potential therapeutic effects of let-7 in two most commonly used murine models of liver fibrosis. Both models have been used in our previous studies [9]. We used AAV8 shown to target hepatocytes but not HSCs and immune cells [[26], [27], [28]]. The AAV8 viruses that express let-7a (AAV8-let-7a) and the control AAV8-vec viruses have been previously described [11].

As shown in Figure 2A, in the CCl_4_ model, mice were administrated with CCl_4_ in addition to AAV8-vec (CCl4 group) or AAV8-let-7a (Let-7a group) at 2 × 10^10^ gc/mouse. In the control group, mice were treated with vehicle and AAV8-vec (Veh group). CCl_4_ (or Veh) was injected intraperitoneally (i.p.) twice a week from week 1 through week 5. AAV8-vec or AAV8-let-7a was injected intravenously once a week from week 3 through week 5. On week 6, mice were sacrificed for sample collection.

As seen in Figure 2B, the Veh group did not develop liver fibrosis, the CCl4 group developed liver fibrosis, and the Let-7a group developed liver fibrosis but significantly less than the CCl4 group. Hydroxyproline is a unique amino acid in collagen molecules and serves as an important biomarker of liver fibrosis. The liver tissue hydroxyproline content was significantly increased in the CCl4 group compared with the Veh group, but the increase was blunted in the Let-7a group (Figure 2C). Blood chemistry analysis revealed elevated alanine transaminase (ALT), aspartate aminotransferase (AST) and bilirubin in the CCl4 group compared with the Veh group, suggesting impaired liver function (Figure 2D). These markers were decreased in the Let-7a group (Figure 2D). When gene expression was analyzed, we observed a decrease in the hepatic let-7a level in the CCl4 group as compared to the Veh group (Figure 2E), consistent with previous reports of decreased let-7 levels in liver tissues from humans and mice with liver fibrosis [18,19]. The hepatic let-7a level increased by ∼2-fold in the Let-7a group as compared to the CCl4 group (Figure 2E). Importantly, there was a significant increase in the expression of *Tet3*, *Fas* and *Tgfb1* both at the mRNA (Figure 2F) and protein (Figure 2G) levels in liver tissues from the CCl4 group compared with the Veh group, but the increase was abolished in liver tissues from the Let-7a group. In parallel, there was an increase in the expression of key fibrotic marker genes *Acta2* and *Col1a1* in the CCl4 group compared with the Veh group, and the increase diminished in the Let-7a group (Figure 2F). Further, there was a marked increase in the number of apoptotic cells in the livers from the CCl4 group compared with the Veh group and Let-7a treatment significantly decreased it (Figure 2H). These results collaborate our in vitro data showing that let-7 inhibits the expression of *TET3*/*Tet3* and *FAS*/*Fas* in obese human and mouse hepatocytes (Figure 1A,C) and that TET3 positively regulates *TGFB1*/*Tgfb1* expression (Figure 1G,I). Taken together, our results show that let-7 is effective in mitigating CCl_4_-induced liver fibrosis likely by simultaneously targeting the TET3/TGF-β and the FAS-mediated pathways in hepatocytes (Figure 1K).

### 3.6 Let-7 mitigates BDL-induced liver fibrosis

In the BDL model, mice were divided into three groups: group 1 (Sham) mice were administrated with AAV8-vec in addition to sham surgery, group 2 (BDL) mice were administrated with AAV8-vec in addition to BDL, and group 3 (Let-7a) mice were administrated with AAV8-let-7a in addition to BDL (Figure 3A). AAV8-vec or AAV8-let-7a was tail vein injected at week 2, 3, and 4. On week 5, mice were sacrificed and blood and tissue samples were harvested.

The Sham mice did not develop liver fibrosis, the BDL mice developed liver fibrosis, the Let-7a mice developed liver fibrosis but significantly less than BDL mice (Figure 3B). As seen in Figure 3C, the liver tissue hydroxyproline content was significantly increased in BDL mice compared with Sham mice, but the increase was abolished in Let-7a mice. Blood chemistry results showed elevated levels of ALT, AST and bilirubin in BDL mice compared with Sham mice, but the levels were reduced in Let-7a mice (Figure 3D). Gene expression analysis showed a 2.5-fold decrease in the hepatic let-7a level in BDL mice and the level was restored to the control level in Let-7a mice (Figure 3E). Meanwhile, there was a significant increase in the expression of *Tet3*, *Fas* and *Tgfb1* both at the mRNA (Figure 3F) and protein (Figure 3G) levels in liver tissues from BDL mice compared with Sham mice, but the increase was blunted in liver tissues from Let-7a mice (Figure 3F,G). In addition, there was an increase in the expression of *Acta2* and *Col1a1* in BDL group compared with Sham group, and the increase diminished in Let-7a mice (Figure 3F). Further, there was a marked increase in the number of apoptotic cells in the livers of BDL mice compared with Sham mice and let-7 treatment significantly decreased it (Figure 3H). Thus, let-7 significantly mitigates liver fibrosis in the BDL model likely by simultaneously targeting the TET3/TGF-β and the FAS-mediated pathways in hepatocytes (Figure 1K).

## 4 Discussion

TGF-β1 released from damaged hepatocytes and activated HSCs acts as key autocrine and paracrine signals to promote fibrogenesis [25]. We have previously identified TET3 as an epigenetic activator of TGF-β1 with the two factors regulating each other in a positive feedback fashion both in hepatocytes and HSCs promoting liver fibrosis [9]. The significance of this regulation was further underscored by our in vivo studies showing that decreasing TET3 expression using siRNAs specifically delivered to the liver of mice using AAV8 viruses ameliorated CCl_4_-induced fibrosis [9]. In the current study, we reveal yet another layer of regulation of TGF-β1 production. On the one hand, we show that let-7 inhibits TET3 expression and that the inhibition is reinforced by a negative feedback loop between the two, leading to decreased production of TGF-β1 from hepatocytes (Figure 1K). On the other hand, let-7 suppresses apoptosis of hepatocytes via the FAS-mediated pathway, thereby indirectly decreasing TGF-β1 production from HSCs (Figure 1K). Hepatocytes make up almost 80 % of the total liver volume whereas non-parenchymal cells contribute only 6.5 % [29]. ROS, hedgehog ligands, nucleotides, lipid peroxides, and cytokines released from apoptotic hepatocytes act as powerful signals that activate HSCs leading to their increased production of TGF-β1 and ECM [25] (Figure 1K). Importantly, this dual action of let-7 (inhibition of both TET3/TGF-β1 and FAS pathways) appears to be conserved in human and mouse hepatocytes (Figure 1). Furthermore, we demonstrate that this newly identified mechanism contributes critically to the anti-fibrogenic effects of let-7 in vivo (Figure 2, Figure 3).

Of note, let-7 fully reversed the increased expression of 5 genes tested by CCL4 and BDL (Figure 2, Figure 3F), but only partially reversed fibrosis (Figure 2, Figure 3B). This suggests that correcting the let-7 pathway identified in this study is likely not sufficient to completely reverse the disease. In addition, a previous study reported that transfection of let-7 into a human hepatic stellate cell line induced apoptosis and decreased mRNA expression of *ACTA2*, *COL1A1* and *COL1A4* (markers of HSC activation) and protein expression of TGF-β, SMAD2 and SMAD3 [18]. However, it remains to be determined whether the regulation of gene expression also occurs in vivo or whether it might be mechanistically connected to the pathogenesis of liver fibrosis.

It has been well documented that let-7 inhibits expression of TET3 and FAS at the post-transcriptional level [[10], [11], [12],[21], [22], [23]]. It is also known that TET3 stimulates TGF-β1 transcription by inducing promoter demethylation [9]. Further, the cellular redox status has long been known to affect the expression of many miRNAs including let-7 [[30], [31], [32]]. For instance, ROS modulates cellular levels of mature miRNAs by regulating the expression and activity of components of the miRNA biogenesis machinery including DGCR8, Drosha, exportin 5 and Dicer [32]. In the current study we have discovered that TET3 also regulates let-7 forming a negative feedback loop between the two (Figure 1K). However, how TET3 regulates let-7 expression is an important question that requires further investigation.

Finally, our discovery of the intrinsic connection between the positive feedback loop of TET3/TGF-β1 and the negative feedback loop of let-7/TET3 helps to explain why inhibiting TET3 or overexpressing let-7 alone have elicited powerful anti-fibrogenic effects in vivo. In light of the success of hepatic targeted gene therapy with AAV vectors in patients with hemophilia B [[33], [34], [35], [36]], we propose that liver-specific delivery of let-7 or its derivatives using AAV vectors holds therapeutic potential for liver fibrosis.

## Author contributions

JS, HL, BL and MH performed experiments, analyzed data, and prepared figures. XZ provided expertise in liver pathology and data interpretation on mouse liver tissue slides. HST provided intellectual insights and critical discussion of the project. YH and DL conceived the project, directed the research and co-wrote the manuscript.

## Declaration of competing interest

The authors declare that they have no known competing financial interests or personal relationships that could have appeared to influence the work reported in this paper.

## Data Availability

The data that has been used is confidential.

## References

[1] Kisseleva T., Brenner D. (2021). Molecular and cellular mechanisms of liver fibrosis and its regression. Nat Rev Gastroenterol Hepatol.

[2] Reinehr R., Haussinger D. (2012). CD95 death receptor and epidermal growth factor receptor (EGFR) in liver cell apoptosis and regeneration. Arch Biochem Biophys.

[3] Schwabe R.F., Tabas I., Pajvani U.B. (2020). Mechanisms of fibrosis development in nonalcoholic steatohepatitis. Gastroenterology.

[4] Ramos-Tovar E., Muriel P. (2020). Molecular mechanisms that link oxidative stress, inflammation, and fibrosis in the liver. Antioxidants.

[5] Tan S., Liu X., Chen L., Wu X., Tao L., Pan X. (2021). Fas/FasL mediates NF-kappaBp65/PUMA-modulated hepatocytes apoptosis via autophagy to drive liver fibrosis. Cell Death Dis.

[6] Wu X., Zhang Y. (2017). TET-mediated active DNA demethylation: mechanism, function and beyond. Nat Rev Genet.

[7] Lio C.J., Yue X., Lopez-Moyado I.F., Tahiliani M., Aravind L., Rao A. (2020). TET methylcytosine oxidases: new insights from a decade of research. J Bio Sci.

[8] Yang J., Bashkenova N., Zang R., Huang X., Wang J. (2020). The roles of TET family proteins in development and stem cells. Development.

[9] Xu Y., Sun X., Zhang R., Cao T., Cai S.Y., Boyer J.L. (2020). A positive feedback loop of TET3 and TGF-beta1 promotes liver fibrosis. Cell Rep.

[10] Cao T., Jiang Y., Wang Z., Zhang N., Al-Hendy A., Mamillapalli R. (2019). H19 lncRNA identified as a master regulator of genes that drive uterine leiomyomas. Oncogene.

[11] Xie D., Chen F., Zhang Y., Shi B., Song J., Chaudhari K. (2022). Let-7 underlies metformin-induced inhibition of hepatic glucose production. Proc Natl Acad Sci U S A.

[12] Li D., Cao T., Sun X., Jin S., Xie D., Huang X. (2020). Hepatic TET3 contributes to type-2 diabetes by inducing the HNF4a fetal isoform. Nat Commun.

[13] Sun B., Karin M. (2012). Obesity, inflammation, and liver cancer. J Hepatol.

[14] Delli Bovi A.P., Marciano F., Mandato C., Siano M.A., Savoia M., Vajro P. (2021). Oxidative stress in non-alcoholic fatty liver disease. An updated Mini review. Front Med.

[15] Arroyave-Ospina J.C., Wu Z., Geng Y., Moshage H. (2021). Role of oxidative stress in the pathogenesis of non-alcoholic fatty liver disease: implications for prevention and therapy. Antioxidants.

[16] Matsuura K., De Giorgi V., Schechterly C., Wang R.Y., Farci P., Tanaka Y. (2016). Circulating let-7 levels in plasma and extracellular vesicles correlate with hepatic fibrosis progression in chronic hepatitis C. Hepatology.

[17] Matsuura K., Aizawa N., Enomoto H., Nishiguchi S., Toyoda H., Kumada T. (2018). Circulating let-7 levels in serum correlate with the severity of hepatic fibrosis in chronic hepatitis C. Open Forum Infect Dis.

[18] Zhang Y., Guo J., Li Y., Jiao K., Zhang Y. (2019). let-7a suppresses liver fibrosis via TGFbeta/SMAD signaling transduction pathway. Exp Ther Med.

[19] Hong W., Li S., Cai Y., Zhang T., Yang Q., He B. (2020). The target MicroRNAs and potential underlying mechanisms of Yiqi-Bushen-Tiaozhi recipe against-non-alcoholic steatohepatitis. Front Pharmacol.

[20] Tag C.G., Weiskirchen S., Hittatiya K., Tacke F., Tolba R.H., Weiskirchen R. (2015). Induction of experimental obstructive cholestasis in mice. Lab Anim.

[21] Yu Y., Liao L., Shao B., Su X., Shuai Y., Wang H. (2017). Knockdown of MicroRNA let-7a improves the functionality of bone marrow-derived mesenchymal stem cells in immunotherapy. Mol Ther.

[22] Geng L., Zhu B., Dai B.H., Sui C.J., Xu F., Kan T. (2011). A let-7/Fas double-negative feedback loop regulates human colon carcinoma cells sensitivity to Fas-related apoptosis. Biochem Biophys Res Commun.

[23] Wang S., Tang Y., Cui H., Zhao X., Luo X., Pan W. (2011). Let-7/miR-98 regulate Fas and Fas-mediated apoptosis. Gene Immun.

[24] Chipman L.B., Pasquinelli A.E. (2019). miRNA targeting: growing beyond the seed. Trends Genet.

[25] Lee Y.A., Wallace M.C., Friedman S.L. (2015). Pathobiology of liver fibrosis: a translational success story. Gut.

[26] Davidoff A.M., Gray J.T., Ng C.Y., Zhang Y., Zhou J., Spence Y. (2005). Comparison of the ability of adeno-associated viral vectors pseudotyped with serotype 2, 5, and 8 capsid proteins to mediate efficient transduction of the liver in murine and nonhuman primate models. Mol Ther.

[27] Rezvani M., Espanol-Suner R., Malato Y., Dumont L., Grimm A.A., Kienle E. (2016). In vivo hepatic reprogramming of myofibroblasts with AAV vectors as a therapeutic strategy for liver fibrosis. Cell Stem Cell.

[28] Carestia A., Kim S.J., Horling F., Rottensteiner H., Lubich C., Reipert B.M. (2021). Modulation of the liver immune microenvironment by the adeno-associated virus serotype 8 gene therapy vector. Mol Ther Methods Clin Dev.

[29] Kmiec Z. (2001). Cooperation of liver cells in health and disease. Adv Anat Embryol Cell Biol.

[30] Lan J., Huang Z., Han J., Shao J., Huang C. (2018). Redox regulation of microRNAs in cancer. Cancer Lett.

[31] Hopkins B.L., Nadler M., Skoko J.J., Bertomeu T., Pelosi A., Shafaei P.M. (2018). A peroxidase peroxiredoxin 1-specific redox regulation of the novel FOXO3 microRNA target let-7. Antioxidants Redox Signal.

[32] Carbonell T., Gomes A.V. (2020). MicroRNAs in the regulation of cellular redox status and its implications in myocardial ischemia-reperfusion injury. Redox Biol.

[33] Nathwani A.C., Reiss U.M., Tuddenham E.G., Rosales C., Chowdary P., McIntosh J. (2014). Long-term safety and efficacy of factor IX gene therapy in hemophilia B. N Engl J Med.

[34] George L.A., Sullivan S.K., Giermasz A., Rasko J.E.J., Samelson-Jones B.J., Ducore J. (2017). Hemophilia B gene therapy with a high-specific-activity factor IX variant. N Engl J Med.

[35] Rangarajan S., Walsh L., Lester W., Perry D., Madan B., Laffan M. (2017). AAV5-Factor VIII gene transfer in severe hemophilia A. N Engl J Med.

[36] Miesbach W., Meijer K., Coppens M., Kampmann P., Klamroth R., Schutgens R. (2018). Gene therapy with adeno-associated virus vector 5-human factor IX in adults with hemophilia B. Blood.

