# Supplementary material for: Let-7 suppresses liver fibrosis by inhibiting hepatocyte apoptosis and TGF-β production
Source: Mol Metab. 2023 Oct 28;78:101828. doi: 10.1016/j.molmet.2023.101828 (PMC10641683; doi:10.1016/j.molmet.2023.101828)
Supplement: Multimedia component 1 [file mmc1.docx]

**Supplementary Information for**

**Let-7 suppresses liver fibrosis by inhibiting hepatocyte apoptosis and TGF-β production**

**Table S1.** qPCR primer sequences

|  | |  | | |  | |  |
| --- | --- | --- | --- | --- | --- | --- | --- |
| **mRNA qPCR primer sequences (mouse)** | | | |  |  | |  |
| **Gene** | | **Forward Primer** | | | **Reverse Primer** | |  |
| Rplp0 | | 5'-GCTCCAAGCAGATGCAGCA-3' | | | 5’-CCGGATGTGAGGCAGCAG-3' | |  |
| Fas | | 5'-TATCAAGGAGGCCCATTTTGC-3' | | | 5'-TGTTTCCACTTCTAAACCATGCT-3' | |  |
| Tgfb1 | | 5'-GGAATACAGGGCTTTCGATT-3' | | | 5'-CTCTGTGGAGCTGAAGCAAT-3' | |  |
| Tet3 | | 5'-GCCGATGCAGTAGTGGAGG-3' | | | 5'-CTGCCTTGAATCTCCATGGTAC-3' | |  |
| Acta2 | | 5'-CTGGCCTAGCAACACTGATT-3' | | | 5'-GTGGACCTTCCTCTGTTGAA-3' | |  |
| Col1a1 | | 5'-ATGATGCTAACGTGGTTCGT-3' | | | 5'-TGGTTAGGGTCGATCCAGTA-3' | |  |
|  | | | |  |  | |  |
| **mRNA qPCR primer sequences (human)** | | | |  |  | |  |
| **Gene** | | **Forward Primer** | | | **Reverse Primer** | |  |
| RPLP0 | | 5'-GGCGACCTGGAAGTCCAACT-3' | | | 5'-CCATCAGCACCACAGCCTTC-3' | |  |
| FAS | | 5'-CCAAGTGACTGACATCAACTC-3' | | | 5'- CTCTTTGCACTTGGTGTTGCTGG-3’ | |  |
| TGFB1 | | 5'-CAAGCAGAGTACACACAGCAT -3' | | | 5'- TGCTCCACTTTTAACTTGAGCC-3' | |  |
| TET3 | | 5'-GACGAGAACATCGGCGGCGT-3' | | | 5'-GTGGCAGCGGTTGGGCTTCT-3' | |  |
|  | | |  | | |  | |
| **Let-7 isoform and U6 qPCR primers (human and mouse)** | | |  | | |  | |
| **Gene** | **Vendor** | | | | | **Catalog number** | |
| Let-7a-2 | Qiagen | | | | | MS00032179 | |
| Let-7b-1 | Qiagen | | | | | MS00001225 | |
| Let-7g-2 | Qiagen | | | | | MS00010983 | |
| RNU6-2-11 | Qiagen | | | | | MS00033740 | |

**Table S2.** Key resources table

| **REAGENT or RESOURCE** | **SOURCE** | | **IDENTIFIER** |
| --- | --- | --- | --- |
| Antibodies | | | |
| Anti-TET3 (for human TET3) | GeneTex | | GTX121453 |
| Anti-TET3 (for mouse TET3) | Active motif | | 61395 |
| Anti-FAS | proteintech | | 60196-1-Ig |
| Anti- TGF-β1 | Proteintech | | 21898-1-AP |
| Anti-GAPDH, HRP-conjugated | Proteintech | | HRP-60004 |
| Anti-rabbit IgG, HRP-linked | Rockland | | 611-1322 |
|  |  | |  |
| Biological Samples | | | |
| Primary human hepatocytes | Discovery Life Sciences | HHA1000-IV210000097060718V1  HHA1000-IV210000118111917V1 | |
| Chemicals, Peptides, and Recombinant Proteins | | | |
| Phosphatase inhibitor cocktail | Thermo | | 78427 |
| Protease inhibitor cocktail | Sigma-Aldrich | | 78438 |
| Dexamethasone | Sigma-Aldrich | | D4902 |
| Insulin | Gibco | | 12585-014 |
| Carbon tetrachloride | Sigma-Aldrich | | 319961 |
| Mineral oil | Sigma-Aldrich | | M5310 |
| Human recombinant fas ligand (FASL) | Sigma-Aldrich | | S8689 |
| Williams Medium | Gibco | | 12551 |
| D-(+)-Glucose | Sigma-Aldrich | | G5767 |
| UCRM media | Discovery Life Sciences | | 81015 |
| UPCM media | Discovery Life Sciences | | 81016 |
| Critical Commercial Assays | | | |
| Bilirubin Assay Kit | Sigma-Aldrich | | MAK126 |
| In Situ Cell Death Detection Kit | Sigma-Aldrich | | 12156792910 |
| One-step TUNEL Assay Kit | Elabscience | | E-CK-A320 |
| Alanine Transaminase Assay Kit | Bioassay Systems | | EALT-100 |
| Aspartate Transaminase Assay Kit | Bioassay Systems | | EASTR-100 |
| Hydroxyproline Assay Kit | Sigma-Aldrich | | MAK008-1KT |
| Lipofectamine RNAiMAX reagent | Invitrogen | | 13778-150 |
| PrimeScript RT Reagent Kit | TaKaRa | | RR037A |
| miScript SYBR Green PCR kit | Qiagen | | 218073 |
| miScript II RT kit | Qiagen | | 218161 |
| SYBR Green PCR Master Mix | Bio-Rad | | 172-5124 |
| PureLink RNA Mini Kit | Ambion | | 12183018A |
| Experimental Models: Organisms/Strains | | | |
| Wild-type C57B/6J male mice | Beijing Vital River Laboratory Animal Technology | | N/A |
| Oligonucleotides and siRNAs | | | |
| Primers for mRNA qPCR see Table S1 | This paper | |  |
| let-7a (let-7a-5p mimic) | Active Motif | | MIM0001 |
| miCon (non-targeting miRNA mimic) | Active Motif | | MIM9001 |
| Viruses | | | |
| AAV8-let-7a (GFPmmu-let-7a-5p AAV serotype 8) | Applied Biological Materials | | Amm1000108 |
| AAV8-vec (empty vector AAV serotype 8) | Applied Biological Materials | | Am00100 |
| Software and Algorithms | | | |
| ImageJ | Schneider et al., 2012 | | https://imagej.nih.gov/ij/ |
| Prism 8 | Graphpad | | https://www.graphpad.com/scientificsoftware/prism/ |
